# Translation, cross-cultural adaptation and validation of the Finnish Diabetes Risk Score (FINDRISC) for use in Brazilian Portuguese: questionnaire validity study

**DOI:** 10.1590/1516-3180.2019.0524.05032020

**Published:** 2020-06-15

**Authors:** Adrianny Larissa Oliveira Conceição, Natália de Castro Corrêa, Patrícia Rodrigues Ferreira, Adriana Sousa Rêgo, Fabricio Brito Silva, Sarah Tarcísia Rebelo Ferreira de Carvalho, Rosane da Silva Dias, Bruna Katarine Beserra Paz, Viviane Chaves de Carvalho Rocha, Daniela Bassi-Dibai

**Affiliations:** I Undergraduate Student, Department of Physical Therapy, Universidade Ceuma (UNICEUMA), São Luís (MA), Brazil.; II Master’s Degree Student, Postgraduate Program on Program Management and Healthcare Services, Universidade Ceuma (UNICEUMA), São Luís (MA), Brazil.; III MSc. Professor, Department of Physical Therapy, Universidade Ceuma (UNICEUMA), São Luís (MA), Brazil.; IV PhD. Professor, Postgraduate Program on Program Management and Healthcare Services, Universidade Ceuma (UNICEUMA), São Luís (MA), Brazil.; V PhD. Professor, Postgraduate Program on Environment, Universidade Ceuma (UNICEUMA), São Luís (MA), Brazil.; VI PhD. Professor, Department of Physical Therapy, Universidade Ceuma (UNICEUMA), São Luís (MA), Brazil.; VII PhD. Coordinator, Postgraduate Program on Program Management and Healthcare Services, Universidade Ceuma (UNICEUMA), São Luís (MA), Brazil.; VIII PhD. Professor, Department of Physical Therapy, Universidade Ceuma (UNICEUMA), São Luís (MA), Brazil.; IX MD, MSc. Professor, Department of Medicine, Universidade Ceuma (UNICEUMA), São Luís (MA), Brazil.; X PhD. Professor, Postgraduate Program on Program Management and Healthcare Services, Universidade Ceuma (UNICEUMA), São Luís (MA), Brazil.

**Keywords:** Surveys and questionnaires, Reproducibility of results, Psychometrics, Public health, Questionnaires, Diabetes, Questionnaire validity

## Abstract

**BACKGROUND:**

The Finnish Diabetes Risk Score (FINDRISC) is a questionnaire that was developed by Finnish researchers to track the risk of diabetes.

**OBJECTIVE:**

To translate, cross-culturally adapt and validate the FINDRISC for use in Brazilian Portuguese.

**DESIGN AND SETTING:**

Questionnaire validity study conducted at a private university.

**METHODS:**

The Brazilian version of the FINDRISC was developed through the processes of translation, back-translation, committee review and pre-testing. Test-retest reliability was measured using the intraclass correlation coefficient (ICC), kappa coefficient, standard error of measurement (SEM) and minimum detectable change (MDC). Internal consistency was measured using Cronbach’s alpha. For construct validity, the total score of the FINDRISC was correlated with the Diabetes Knowledge Scale (DKN-A) and Diabetes Mellitus Risk Questionnaire (QRDM). Ceiling and floor effects were also evaluated in the present study.

**RESULTS:**

For construct validity and floor and ceiling effect measurements, a total sample of 107 participants was used. For reliability, a subsample of 51 participants out of the total sample was used. We identified adequate values for reliability (kappa ≥ 0.79 and ICC = 0.98) and internal consistency (Cronbach’s alpha = 0.84). Regarding the error inherent in the FINDRISC, we found SEM = 8.02% and MDC = 22.44%. There were significant correlations between the FINDRISC and the QRDM (r = 0.686) and DKN-A (r = -0.216). No ceiling or floor effects were found.

**CONCLUSION:**

The Brazilian version of the FINDRISC has adequate psychometric properties that are in accordance with the best international recommendations.

## INTRODUCTION

Recently, the International Diabetes Federation reported that the number of individuals with type 2 diabetes mellitus (T2DM) had increased worldwide, especially in Brazil, which placed this country in fifth place among the countries with the most people with diabetes mellitus.^1^

Given this reality, strategies are being sought in an attempt to easily and cost-effectively screen individuals with high potential to develop T2DM, in order to implement preventive measures against the onset of the disease. In this context, use of questionnaires has been an ally in screening for several other diseases.^2^^,^^3^^,^^4^^,^^5^

A few questionnaires directed towards people with T2DM have been validated for Brazilian Portuguese, including the Diabetes Quality of Life Measure,^6^ Diabetes Mellitus Knowledge and Attitude Questionnaire^5^ and Diabetes Self-Care Activities.^7^ With regard to the risk of developing T2DM, there is no validated questionnaire for use in Brazilian Portuguese for these purposes.

The Finnish Diabetes Risk Score (FINDRISC) is a questionnaire that was developed in Finnish and English in 2003 by Finnish researchers. It had the aims of tracking the risk of diabetes and stimulating the adoption of measures to prevent the onset of T2DM, especially for individuals who are at increased risk of the disease, but without the need for low-cost laboratory tests.^8^ Some articles using the FINDRISC have already been published in Brazilian Portuguese,^9^^,^^10^ even without proper translation, cross-cultural adaptation or validation of the questionnaire for this language. However, the FINDRISC has already been validated for other languages, including those spoken by the populations of Spain,^11^ Greece,^12^ Venezuela,^13^ Colombia,^14^ Hungary,^15^ Germany,^16^ Jordan,^17^ China,^18^ Norway^19^ and Slovenia.^20^

## OBJECTIVE

Considering the importance of instruments that track the risk of developing T2DM, for the context of public health, the aim of this study was to translate, cross-culturally adapt and validate the FINDRISC for use in Brazilian Portuguese.

## METHODS

### Study design and ethics

This was a cross-sectional study on the translation, cross-cultural adaptation and validation of a questionnaire. It was conducted in accordance with the Guidelines for the Process of Cross-Cultural Adaptation of Self-Report Measures^21^ and the COnsensus-based Standards for the selection of health Measurement INstruments (COSMIN).^22^ Authorization for analysis of the psychometric properties of the FINDRISC in Brazilian Portuguese was granted via e-mail by the authors of the original version of the questionnaire (Dr. Jaana Lindstrom and Dr. Jaakko Tuomilehto).

This study was approved by the Research Ethics Committee of our institution, under number 2.853.570, on August 29, 2018. All the participants validated their participation by signing a free and informed consent statement. These participants were recruited from the university community in the city of São Luís (MA), Brazil, and from a community associated with this city. Announcements regarding this study were disseminated through pamphlets and social media.

### FINDRISC translation and cross-cultural adaptation

The process of translation and cross-cultural adaptation of the FINDRISC for use in Brazilian Portuguese followed the criteria of Beaton et al.^21^ and was performed in five phases as described below:

1. Translation: two independent translators, both with Brazilian Portuguese as their mother tongue and fluent in English, translated the original version of the questionnaire into Brazilian Portuguese;
2. Synthesis of translations: after discussions and revisions, the two translators, under observation of one of the researchers, produced a synthesis from the two versions of the independently translated questionnaire, thus resulting in a combined version in a consensual manner;
3. Back-translation: two independent translators (without technical knowledge of health issues), both with English as the mother tongue and fluent in Portuguese, translated the Portuguese version of the questionnaire back to English, without previous knowledge of the original version of the questionnaire;
4. Expert committee review: six diabetes specialists, along with the four translators of this study, reviewed the original version and the translated, synthetized and back-translated versions, and defined the pre-final version of the FINDRISC;
5. Pre-final version test: the pre-final version of the questionnaire was administered to 30 individuals without a diagnosis of diabetes and with Portuguese as their mother tongue. The respondents’ comprehension of the items and responses in the FINDRISC was evaluated.

### Participants

The sample size for this validation study was based on COSMIN, and a minimum of 100 individuals was recommended.^23^ The eligibility criteria that we used were that the participants could be of either sex, without any diagnosis of type 1 or type 2 diabetes mellitus, aged over 24 years and under 64 years, and without cognitive deficits or any other limitations that would make it impossible for them to respond to the questionnaire.

## FINDRISC

The FINDRISC consists of eight items that investigate and rate the risk of developing T2DM within 10 years. The responses to each item are scored according to their influence on the development of the disease. The total score can range from 0 to 26 points and is classified as follows: ≤ 7 points, low risk (1 in 100 people will develop disease); 7 to 11 points, slightly elevated risk (1 in 25 people will develop the disease); 12 to 14 points, moderate risk (1 in 6 people will develop the disease); 15 to 20 points, high risk (1 in 3 people will develop the disease); and > 20 points, very high risk (1 in 2 people will develop the disease).

## OTHER QUESTIONNAIRES

In addition to the FINDRISC, we applied two other questionnaires that had already been adapted and validated for use in Brazilian Portuguese, to ascertain the construct validity. These instruments were:

1. Diabetes Knowledge Scale (DKN-A), a questionnaire that was validated for the Brazilian population by Torres et al.,^5^ which is composed of 15 multiple-choice questions on various aspects of general knowledge relating to T2DM. The total score is calculated by assigning one point to each correct answer, and it can range from 0 to 15. The higher the score is, the greater the respondent’s knowledge about T2DM is.
2. Diabetes Mellitus Risk Questionnaire (QRDM), a questionnaire that was validated in the master’s degree dissertation of Cruz,^3^ which is composed of seven items, with a total score that can range from 0 to 27 points. The higher the score is, the higher the respondent’s risk of developing T2DM is.

### Statistical analysis

Reliability was assessed based on a test-retest model by measuring the kappa coefficient, intraclass correlation coefficient (ICC), standard error of measurement (SEM) and minimum detectable change (MDC). Internal consistency was assessed using Cronbach’s alpha. The kappa values were interpreted based on the categories defined by Sim and Wright: < 0, poor; 0.01-0.20, slight; 0.21-0.40, fair; 0.41-0.60, moderate; 0.61-0.80, substantial; and 0.81-1, almost perfect.^24^ The ICC values were interpreted based on the study by Fleiss: for values below 0.40, reliability was considered low; between 0.40 and 0.75, moderate; between 0.75 and 0.90, substantial; and greater than 0.90, excellent.^25^ The SEM percentage was interpreted based on the definitions of Ostelo et al.: 5% or less, very good; greater than 5% and less than or equal to 10%, good; greater than 10% and less than or equal to 20%, doubtful; and greater than 20%, negative.^26^

To ascertain the validity of the construct, Pearson’s correlation coefficient (r) was used to determine the magnitude of the correlations between the FINDRISC and the QRDM, and between the FINDRISC and the DKN-A. The r values were interpreted in accordance with the COSMIN recommendations: correlations with instruments measuring similar constructs should be ≥ 0.50; correlations with instruments measuring related but dissimilar constructs should be 0.30-0.50; and correlations with instruments measuring unrelated constructs should be < 0.30.^22^

Ceiling and floor effects were evaluated in the present study. By definition, these effects occurred when a number of study participants (set as over 15%) reached the minimum or maximum values of the total score of the questionnaire.

## RESULTS

### Sample characterization

The study consisted of three samples of different sizes. Initially, for the analysis on the pre-test version of the instrument, 30 participants were included. For the assessment of construct validity and measurement of the floor and ceiling effects, a total sample consisting of 107 participants who were evaluated at a single time was used. For determine the reliability of the FINDRISC, a subsample was used, comprising 51 participants from the total sample who were evaluated at two times (test and retest), seven days apart. The sample size used in this validation study was in accordance with international practices for cross-cultural adaptation and validation of questionnaires, as guided by the study of Beaton et al.^21^ and by COSMIN.^22^

The participants’ characteristics are described in Table 1, along with the mean scores from the QRDM and DKN-A questionnaires.

**Table 1.** Characteristics of study participants according to study phase

Characteristic	Reliability phase (n = 51)	Validity phase (n = 107)
Gender (female)^a^	38 (74.51%)	80 (74.8%)
Age (years)^b^	36.90 (10.67)	42.88 (12.35)
Schooling^a^		
Elementary education	3 (5.9%)	18 (16.9%)
High school	30 (58.8%)	59 (55.1%)
Higher education	18 (35.3%)	30 (28%)
Marital status^a^		
Single	35 (68.6%)	60 (56.1%)
Married	14 (27.4%)	43 (40.2%)
Divorced	1 (2.0%)	2 (1.9%)
Widowed	1 (2.0%)	2 (1.9%)
Height (m)^b^	1.59 (0.24)	1.59 (0.18)
Weight (kg)^b^	67.62 (12.18)	68.09 (12.61)
BMI (kg/m^2^)^b^	25.58 (3.93)	26.26 (4.46)
Abdominal circumference (cm)^b^	84.52 (12.00)	87.48 (12.48)
QRDM (score)^b^	7.80 (5.16)	9.27 (5.30)
DKN-A (score)^b^	7.94 (2.58)	7.63 (2.69)

^a^Values presented as absolute number (percentage); ^b^Values presented as mean (standard deviation).

BMI = body mass index; QRDM = Diabetes Mellitus Risk Questionnaire; DKN-A = Diabetes Knowledge Scale.

### Translation and cross-cultural adaptation

The translation and back-translation processes are described in Table 2. After the two translations performed by Portuguese native speakers, the translations were synthetized, and the translators came to an agreement regarding the definitions for the eight items of the FINDRISC in Brazilian Portuguese. Next, back-translation was performed by two English native speakers. After this phase, there was a meeting involving the translators and a committee of experts, and the synthetized version was accepted unanimously, without any amendments proposed. In this manner, the pre-final version of the FINDRISC was defined.

**Table 2.** Translation, consensus version and backtranslation of Finnish Diabetes Risk Score (FINDRISC)

Original version of FINDRISC		Translation	Consensus version	Backtranslation
Number	Item			
1	Age.	T1: Idade. T2: Idade.	T12: Idade.	B1: Age. B2: Age.
2	Body mass index.	T1: Índice de massa corporal (IMC). T2: Índice de massa corporal.	T12: Índice de massa corporal (IMC).	B1: Body mass index (BMI). B2: Body mass index (BMI).
3	Waist circumference measured below the ribs (usually at the level of the navel).	T1: Circunferência da cintura medida abaixo das costelas (geralmente na altura do umbigo). T2: Medida da cintura (geralmente ao nível do umbigo).	T12: Circunferência da cintura medida abaixo das costelas (geralmente na altura do umbigo).	B1: Waist circumference measured below the ribs (generally at the height of the navel). B2: Waist circumference measured below the ribs (generally at height of belly button).
4	Do you usually have daily at least 30 minutes of physical activity at work and/or during leisure time (including normal daily activity)?	T1: Você costuma ter pelo menos 30 minutos de atividade física diária no trabalho e/ou durante o horário de lazer (incluindo as atividades diárias normais)? T2: Pratica, diariamente, atividade física pelo menos durante 30 minutos, no trabalho ou durante o tempo livre (incluindo atividades da vida diária)?	T12: Você pratica pelo menos 30 minutos de atividade física diária no trabalho e/ou durante o horário de lazer (incluindo as atividades diárias normais)?	B1: Do you practice at least 30 minutes of daily physical activity at work and/or during leisure time (including normal daily activities)? B2: Do you practice at least 30 minutes of daily physical activity at work and/or during leisure time (including normal daily activities)?
5	How often do you eat vegetables, fruit or berries?	T1: Com que frequência você come legumes e verduras, frutas ou grãos? T2: Com que frequência você consome vegetais ou frutas?	T12: Com que frequência você come legumes, verduras, frutas ou grãos?	B1: How often do you eat legumes, vegetables, fruit or grains? B2: How often do you eat vegetables, fruits, or grains?
6	Have you ever taken medication for high blood pressure on regular basis?	T1: Você já tomou alguma medicação para pressão alta regularmente? T2: Já tomou regularmente algum medicamento para pressão alta?	T12: Você já tomou regularmente algum medicamento para pressão alta?	B1: Do you regularly take any medication for high blood pressure? B2: Have you ever taken any medication for high blood pressure?
7	Have you ever been found to have high blood glucose (e.g. in a health examination, during an illness, during pregnancy)?	T1: Alguma vez você já apresentou glicose alta no sangue (exemplo: em um exame médico, durante uma doença, durante gravidez)? T2: Alguma vez teve açúcar elevado no sangue (por ex: em exame de rotina, durante alguma doença, na gravidez)?	T12: Alguma vez você já apresentou glicose alta no sangue (por exemplo, em um exame médico de rotina, durante uma doença, durante gravidez)?	B1: Have you ever had high blood glucose (for example, at a routine medical examination, during an illness, during pregnancy)? B2: Have you ever had high blood glucose (for example, during a routine medical examination, during illness, during pregnancy)?
8	Have any of the members of your immediate family or other relatives been diagnosed with diabetes (type 1 or type 2)?	T1: Algum membro de sua família imediata ou outro parente já foi diagnosticado(a) com diabetes (tipo 1 ou tipo 2)? T2: Algum membro da sua família ou parente próximo já foi diagnosticado com diabetes (tipo 1 ou 2)?	T12: Algum membro de sua família ou parente próximo já foi diagnosticado com diabetes (tipo 1 ou tipo 2)?	B1: Has any member of your family or close relative ever been diagnosed with diabetes (type 1 or type 2)? B2: Has any member of your immediate family or relative ever been diagnosed with diabetes (type 1 or type 2)?

T1 = translation 1; T2 = translation 2; T12 = consensual synthesis of translations 1 and 2; B1 = backtranslation 1; B2 = backtranslation 2.

The pre-final version of the FINDRISC was applied to 30 individuals (24 women, 80%) without a diagnosis of diabetes and with Brazilian Portuguese as their mother tongue. The average age of this sample was 49 years (standard deviation, SD = 10.82), and the average FINDRISC score was 11.56 points (SD = 4.70). There was 100% understanding of the questionnaire items, which thus defined the final version of the FINDRISC in Brazilian Portuguese (Appendices 1 and 2).

### Reliability and internal consistency

Table 3 presents the reliability values item-by-item and the total score of the final version of the FINDRISC in Brazilian Portuguese. We found adequate values for reliability (kappa ≥ 0.66 and ICC = 0.98) and internal consistency (Cronbach’s alpha ≥ 0.82). In addition, in calculating the error inherent to the FINDRISC total score, we found that the values were sufficient, as follows: SEM (absolute) = 0.63; SEM (%) = 8.02; MDC (absolute) = 1.76; and MDC (%) = 22.44.

**Table 3.** Finnish Diabetes Risk Score (FINDRISC) reliability and internal consistency with presentation of mean values, standard deviation (SD), kappa or intraclass correlation coefficient (ICC) with 95% confidence interval (CI) and Cronbach’s alpha

FINDRISC item	Mean (SD)		Reliability	Cronbach’s alpha if item excluded
	Test	Retest		
1	0.58 (1.10)	0.56 (1.10)	kappa = 1.00 (95% CI = 1.00, 1.00)	0.83
2	0.88 (1.03)	0.88 (1.06)	kappa = 1.00 (95% CI = 1.00, 1.00)	0.83
3	1.96 (1.76)	1.90 (1.78)	kappa = 0.90 (95% CI = 0.85, 0.95)	0.82
4	1.01 (1.00)	1.17 (0.99)	kappa = 0.68 (95% CI = 0.61, 0.74)	0.84
5	0.60 (0.80)	0.54 (0.50)	kappa = 0.72 (95% CI = 0.65, 0.78)	0.84
6	0.11 (0.47)	0.11 (0.47)	kappa = 1.00 (95% CI = 1.00, 1.00)	0.84
7	0.09 (0.70)	0.19 (0.98)	kappa = 0.66 (95% CI = 0.46, 0.86)	0.84
8	2.70 (2.10)	2.70 (2.10)	kappa = 1.00 (95% CI = 1.00, 1.00)	0.83
Total score	7.82 (4.32)	8.01 (4.66)	ICC = 0.98 (95% CI = 0.97, 0.99)	Cronbach’s alpha = 0.84

### Construct validity

The construct validity was tested by correlating the total FINDRISC score with the scores from the other questionnaires of the present study. The following significant (P < 0.05) and adequate correlations with this validation criterion were observed: QRDM (r = 0.686) and DKN-A (r = -0.216). According to COSMIN, values ≥ 0.50 are expected for correlations of questionnaire scores with similar constructs (as in the case of QRDM) and values < 0.30 for correlations of questionnaire scores with unrelated constructs (as in the case of DKN-A).

### Ceiling and floor effects

Two individuals (1.9%) achieved a FINDRISC minimum score of 0. No participant reached the maximum score of 26 points. Therefore, ceiling and floor effects were not observed.

## DISCUSSION

We observed that the FINDRISC in Brazilian Portuguese presented an adequate level of comprehension in the study population and acceptable values for reliability, internal consistency and validity. Regarding reliability, our study found kappa values (when considered item-by-item) ranging from 0.66 to 1.00 and ICC values (when considering the total score) of 0.98. In addition, Cronbach’s alpha of 0.84 was found. Significant correlations were found between the FINDRISC and DKN-A and between the FINDRISC and QRDM.

The FINDRISC has also been considered to be a valid questionnaire in other countries. However, these validations did not have cross-cultural translation and adaptation, and only tested the accuracy of diagnosing the risk of developing diabetes based on FINDRISC cutoff points. All of these studies used the receiver operating characteristic (ROC) curve as a statistical method. Thus, the following findings were observed: area under the ROC curve of 0.77 in Norway,^19^ greater than 0.70 in Venezuela, of 0.78 in Slovenia,^20^ of 0.74 in Mexico,^27^ of 0.76 in China,^18^ greater than 0.72 in Greece^12^ and greater than 0.74 in Spain.^11^ It is noteworthy that the values for the area under the ROC curve that were identified in these various studies established that the degree of accuracy of the FINDRISC was adequate.

The international best practices for translation, cross-cultural adaptation and validation of questionnaires are centered on COSMIN.^22^ These methodological guidelines indicate several psychometric properties that an instrument should present, with emphasis on i) reliability and ii) validity (composed of several subitems, such as face, content, construct, structural, cross-cultural and criterion validity) and responsiveness. Our validation study involved assessments of reliability (using kappa, ICC, SEM and MDC), cross-cultural adaptation (using translation, synthesis of translations, back-translation, expert committee and pre-final testing), construct validity (using the correlation between questionnaires) and structural validity (using Cronbach’s alpha). These properties ensured that the FINDRISC can be applied to the Brazilian population. Other questionnaire validation studies conducted in Brazil have also measured these psychometric properties.^28^^,^^29^^,^^30^

Some limitations of this study and suggestions need to be noted. Firstly, we recommend that the cross-cultural adaptation of the FINDRISC to other languages should be tested based on COSMIN.^22^ Lack of such testing greatly limited the discussion of the results. In addition, because of the need for specific methodology, our study did not investigate the accuracy or responsiveness of the FINDRISC. Thus, we suggest that future studies should measure these psychometric properties in Brazilian Portuguese.

## CONCLUSION

The results from this study demonstrate that the Brazilian Portuguese version of the FINDRISC has adequate psychometric properties that are in accordance with the best international recommendations. Thus, its use within clinical routines and/or research can be supported.
